# Barriers to Accessing Primary Care and Appropriateness of Healthcare Among Immigrants in Italy

**DOI:** 10.3389/fpubh.2022.817696

**Published:** 2022-02-09

**Authors:** Anteo Di Napoli, Martina Ventura, Teresa Spadea, Paolo Giorgi Rossi, Letizia Bartolini, Laura Battisti, Laura Cacciani, Nicola Caranci, Achille Cernigliaro, Marcello De Giorgi, Antonio Fanolla, Marco Lazzeretti, Mariangela Mininni, Concetta Mirisola, Alessio Petrelli

**Affiliations:** ^1^National Institute for Health, Migration and Poverty (INMP), Roma, Italy; ^2^Epidemiology Unit, Local Health Unit TO3 Piedmont Region, Grugliasco, Italy; ^3^Epidemiology Unit, Azienda Unità Sanitaria Locale – Istituto di Ricovero e Cura a Carattere Scientifico di Reggio Emilia, Reggio Emilia, Italy; ^4^Epidemiological Observatory, Public Health Department - Local Health Unit, Trento, Italy; ^5^Department of Epidemiology of the Lazio Regional Health Service, Roma, Italy; ^6^Regional Health and Social Care Agency, Bologna, Italy; ^7^Health Authority Sicily Region and Local Authority Trapani Province, Palermo, Italy; ^8^Umbria Digitale - Health Information and Communication Technology Service, Regional Health Authority of Umbria, Perugia, Italy; ^9^Provincial Government South Tyrol, Observatory for Health, Bolzano, Italy; ^10^Tuscany Regional Health Agency, Florence, Italy; ^11^Primary Prevention Office, People Policies Department, Potenza, Italy

**Keywords:** immigrants, access, intervention policies, equity, monitoring system

## Abstract

**Introduction:**

The health status and health care needs of immigrant populations must be assessed. The aim of this study was to evaluate barriers to accessing primary care and the appropriateness of health care among resident immigrants in Italy, using indicators regarding maternal health, avoidable hospitalization, and emergency care.

**Methods:**

Cross-sectional study using some indicators of the National Monitoring System of Health Status and Healthcare of the Immigrant Population (MSHIP), coordinated by the National Institute for Health, Migration and Poverty (INMP), calculated on perinatal care, hospital discharge, and emergency department databases for the years 2016–2017 in nine Italian regions (Piedmont, Trento, Bolzano, Emilia-Romagna, Tuscany, Umbria, Latium, Basilicata, Sicily). The analyses were conducted comparing immigrant and Italian residents.

**Results:**

Compared to Italian women, immigrant women had fewer than five gynecological examinations (8.5 vs. 16.3%), fewer first examinations after the 12th week of gestational age (3.8 vs. 12.5%), and fewer than two ultrasounds (1.0 vs. 3.8%). Compared to Italians, immigrants had higher standardized rates (× 1,000 residents) of avoidable hospitalizations (males: 2.1 vs. 1.4; females: 0.9 vs. 0.7) and of access to emergency departments for non-urgent conditions (males: 62.0 vs. 32.7; females: 52.9 vs. 31.4).

**Conclusions:**

In Italy, there appear to be major issues regarding accessing services and care for the immigrant population. Policies aimed at improving socioeconomic conditions and promoting integration can promote healthy lifestyles and appropriate access to health care, counteracting the emergence of health inequities in the immigrant population.

## Introduction

Migration flows from developing countries toward Europe, both for economic reasons and to flee war and persecution, have increased in the last few decades, in particular toward Mediterranean countries (1). As a result, the social and economic impact of international migration has become one of the most pressing and debated issues in recent years. Until the 1980s, immigration to Italy was not a significant phenomenon. However, the number of immigrants has been increasing since then, with Italy fully becoming a destination country of immigration in the last 20 years (1).

The number of resident immigrants in Italy has stabilized at around 5 million in recent years: on 1 January 2020, there were 5,039,637 resident immigrants, equal to 8.5% of the total resident population, almost double that in 2005 (~2,400,000, 4.1% of total resident population). Of this 2020 resident immigrant population, 52% were female, and the most frequent countries of origin were Romania (23%), Albania (8%), Morocco (8%), China (6%), and Ukraine (5%) (2).

Unsurprisingly, immigration has generated new cultural and economic challenges in Italy, in particular for its health-care system (3–6). Each European country manages the integration of its immigrant populations differently, developing its own policies regarding family reunification, education, political participation, long-term residence, access to nationality, anti-discrimination laws, and labor market mobility (7, 8). In terms of responding to the health needs of immigrant populations, it is well-known that this can be complex due to cultural, religious, and linguistic differences, to these individuals' exposure to specific risk factors before, during, and after migration (3, 9, 10), and to their attitude toward interacting with the health system of the host country(11, 12).

While immigrants are generally healthier than native-born residents due to a phenomenon known as the “healthy migrant effect,” the selection of individuals in better health who can undertake and cope with the migratory process, they are nevertheless a potentially vulnerable population. Their adverse health outcomes are often preventable and can be explained by the substantial barriers to accessing preventive health care, such as pre-natal and maternal healthcare, by a lack of awareness of their rights and of the provision of health care in the host country, by linguistic and cultural issues, and by financial limitations (13–15).

According to the WHO, the right to health implies equal and timely access to health-care services for equal health needs, the provision of health-related education and information, and the participation of the population in health-related decisions at the national and community levels. Health-care services should be physically and financially accessible to all subgroups of the population, including vulnerable groups, and should be delivered without any discrimination (16, 17).

In Italy, despite the fact that access to health and social services is guaranteed to the immigrant population through the universal health care provided by the Italian National Health System, there are strong social and territorial inequalities in guaranteeing essential levels of care (6, 18). It is therefore essential to assess the health status and health-care needs of this population. To this end, the National Monitoring System of Health Status and Healthcare of the Immigrant Population (MSHIP), coordinated by the National Institute for Health, Migration and Poverty (INMP) (19, 20), conducts systematic, timely monitoring of the health status and health care of the resident immigrant population in Italy.

The comparison of the data collected by the MSHIP on Italians and immigrants in terms of their health status and the assistance they receive has revealed major issues particularly in three areas: access to maternal health care, use of emergency/urgent care, and hospitalization for conditions that could be cared for or prevented by appropriate outpatient services (19). These findings are consistent with studies conducted in other European countries (21–25).

The aim of the present study was to compare the use of maternal health care and of emergency care and the occurrence of avoidable hospitalizations in immigrants and in Italians through the analysis of routinely collected indicators to determine whether barriers to immigrants' accessing primary care in Italy result in inappropriate care.

## Methods

This cross-sectional study of the Italian and foreign populations residing in Italy in 2016–2017 used a subset of the health and health-care indicators of the MSHIP. The complete set of MSHIP indicators was developed through a project funded by the Italian Ministry of Health that involved experts from the main research institutes involved in this field and stakeholders from the national and regional health services. The indicators were developed in order to monitor all the health issues concerning migrant health (26). In the present study, to address the issues of maternal health care, emergency/ urgent care, and avoidable hospitalizations, the MSHIP Working Group selected those indicators for which reliable data were available for the entire study period and for the geographic areas.

This subset included indicators on perinatal care, hospital discharge, and emergency care. The study period included the years 2016 and 2017; data were collected from nine regions and autonomous provinces, covering about 41% of the resident population in Italy and 45% of the foreign population. Four of the nine regions and autonomous provinces (Piedmont, Trento, Bolzano, Emilia-Romagna) are in Northern Italy, 3 (Tuscany, Umbria, Latium) in Central Italy, and 2 (Basilicata, Sicily) in Southern Italy.

We chose to use the term *immigrant* to define foreign citizens, including those born in Italy, with indicator-specific age restrictions.

The main outcome measures investigated referred to three areas:

(1) Maternal health: number and appropriate schedule of prenatal visits, number of ultrasounds.

(2) Avoidable hospitalizations (admissions that could have been avoided due to having one or more of a subset of conditions that could have been prevented or treated outside of the inpatient setting) (27): standardized rates of avoidable hospitalizations.

(3) Emergency care: access to an emergency department by triage code, which in Italy is based on four priority levels: red (life-threating conditions), yellow (potentially life-threatening conditions), green (minor injuries or illnesses), and white (non-urgent conditions).

### Statistical Analysis

Baseline characteristics of the study populations by citizenship were evaluated. Indicators of maternal health are presented as proportions, and *p*-values of the differences between Italians and immigrants were calculated.

For avoidable hospitalization, the analyses were restricted to individuals aged 20–64 years to exclude the pediatric population and the older age classes, in which immigrants in Italy are highly underrepresented.

For age-standardized rates of assignment of triage codes at access to the emergency department, the analyses were restricted to individuals aged <65 years to exclude the older age classes, in which immigrants in Italy are highly underrepresented.

The indicators presented as standardized rates were calculated using the direct standardization method, using as the reference population that resident in Italy on 1 January 2018. The 95% confidence intervals (CIs) for standardized rates were calculated.

## Results

The population of the regions and autonomous provinces that participated in the MSHIP included 2,410,645 immigrants (males: 1,142,867; females: 1,267,778) and 23,650,057 Italian citizens (males: 11,482,763; females: 12,625,630), equal to 42.6% and to 47.8% of the population residing in the national territory, respectively. The proportion of immigrants in the MSHIP population was slightly higher than in the population of the residents in Italy (8.3%).

### Maternal Health Care

In the 2 years considered, 24.1% of deliveries involved immigrant women, who were on average younger than Italian women (30.3 vs. 33.3), more frequently had a low education level (45.7 vs. 23.3%) and were more often married (64.9 vs. 53.2%) (data not shown).

Table 1 shows the distribution of some indicators of maternal health-care access by citizenship.

**Table 1:** Distribution of indicators of maternal health care access by citizenship.

**Pregnancies**	**Italians**		**Immigrants**	
	***N***	**%**	***N***	**%**
	259,011	75.9	82,142	24.1
Less than 5 visits during pregnancy	22,016	8.5	13,389	16.3
First visit at more than 12 weeks of gestational age	9,842	3.8	10,268	12.5
Less than 2 ultrasounds during pregnancy	2,642	1.0	3,121	3.8

*Italy, MSHIP, 2016–2017*.

The percentage of immigrant women who received fewer than five gynecological visits during pregnancy was about twice that of Italian women (16.3 vs. 8.5%; *p* < 0.01). The percentage of immigrant women who had their first prenatal visit after 12 weeks of gestational age was more than three times that of Italians (12.5 vs. 3.8%; *p* < 0.01). The percentage of immigrant women who underwent fewer than two ultrasounds in pregnancy was about four times that of Italians (3.8 vs. 1.0%; *p* < 0.01). The percentage of immigrant women who underwent invasive prenatal testing after age 35 was about one third that of Italians (3.0 vs. 8.9%; *p* < 0.01).

### Avoidable Hospitalization

Table 2 shows proportions and age-standardized rates per 1,000 residents of avoidable hospitalizations, by sex and citizenship among subjects aged 20–64 years.

**Table 2:** Avoidable hospitalizations: proportions and age-standardized rates per 1,000 residents, by sex and citizenship (20–64 years).

	**Italians**				**Immigrants**			
**Avoidable**	***N***	**%**	**Age-standardized**	**95% CI**	***N***	**%**	**Age-standardized**	**95% CI**
**hospitalizations**			Rate*1,000				Rate*1,000	
Males	18,213	89.3	1.43	(1.41–1.45)	2,176	10.7	2.14	(2.04–2.23)
Females	8,427	86.6	0.67	(0.66–0.69)	1,302	13.4	0.92	(0.87–0.97)
Total	26,640	88.5	1.06	(1.04–1.07)	3,478	11.5	1.41	(1.36–1.46)

*Comparison between proportions of Italians and immigrants: p < 0.01*.

*Italy, MSHIP, 2016–2017*.

In the 2 years considered in the participating regions, the age standardized rate of avoidable hospitalization was higher among immigrants than Italians both for males (2.14 vs. 1.43) and females (0.92 vs. 0.67).

### Access to Emergency Care

The total number of accesses to emergency departments among immigrant subjects aged <65 years was 1,958,276 (52.3% females) and was 14,673,559 (50.1% females) among Italians (data not reported in table).

Table 3 shows proportions and age-standardized rates per 1,000 residents of emergency care access, by triage code, sex, and citizenship.

**Table 3:** Emergency care access: proportions and age-standardized rates per 1,000 residents, by triage code, sex, and citizenship (<65 years).

	**Italians**				**Immigrants**			
**Triage**	***N***	**%**	**Age-standardized**	**95% CI**	***N***	**%**	**Age standardized**	**95% CI**
**code**			Rate*1,000				Rate*1,000	
**Males**	7,323,122	88.7	309.2	(308.9–309.5)	933,614	11.3	371.8	(370.9–372.7)
White	692,919	9.5	32.7	(32.6–32.8)	152,510	16.3	62.0	(61.6–62.4)
Green	4,808,459	65.7	221.3	(221.1–221.6)	633,055	67.8	252.7	(252.0–253.5)
Yellow	1,660,815	22.7	51.5	(51.4–51.6)	136,498	14.6	52.7	(52.3–53.1)
Red	160,929	2.2	3.7	(3.7–3.7)	11,551	1.2	4.4	(4.3–4.5)
**Females**	7,350,437	87.8	299.4	(299.2–299.7)	1,024,662	12.2	365.3	(364.5–366.1)
White	673,432	9.2	31.4	(31.3–31.5)	141,033	13.8	52.9	(52.651–53.2)
Green	4,898,299	66.6	219.4	(219.1–219.6)	717,676	70.0	257.6	(257.0–258.3)
Yellow	1,642,992	22.4	46.6	(46.5–46.7)	157,686	15.4	52.3	(52.0–52.6)
Red	135,714	1.8	2.1	(2.1–2.1)	8,267	0.8	2.5	(2.4–2.6)

*Comparison between proportions of Italians and immigrants: p < 0.01*.

*Italy, MSHIP, 2016–2017*.

The age-standardized rates were always higher among immigrants than among Italians, both for males (371.8 vs. 309.2) and females (365.3 vs. 299.4). These findings were confirmed for each triage code: white (62.0 vs. 32.7), green (252.7 vs. 221.3), yellow (52.7 vs. 51.5), red (4.4 vs. 3.7) among males; white (52.9 vs. 31.4), green (257.6 vs. 219.4), yellow (52.3 vs. 46.6), red (2.5 vs. 2.1) among females.

For all the indicators considered, a strong variability between regions was observed (Supplementary Tables S1–S4).

## Discussion

Our study shows that among immigrants residing in Italy, there is overall less access to appropriate maternal health care. During pregnancy, a higher frequency of first visits after the 12th week of pregnancy and a lower frequency of the recommended number of prenatal gynecological examinations and ultrasounds and of invasive prenatal tests after the age of 35 were observed. This evidence is all the more worrisome when one considers that immigrant women account for approximately one quarter of all births and have a 70% excess mortality in newborns compared to Italians (22).

These results suggest that foreign women receive worse health care than do Italians in terms of adherence to the recommendations on examinations and ultrasounds. Despite the universal health care provided by the Italian NHS, immigrant women still face problems accessing maternal care (28–31), probably related to administrative, linguistic, and/ or cultural barriers (23, 30).

The frequency of day-hospital admissions of females in childbearing age, which was decidedly higher among immigrants than among Italians (67 vs. 37%), suggests there were delays and problems in accessing maternal care during pregnancy (19).

Another finding was that the standardized rate of avoidable hospitalization was higher among immigrants than among Italians by about 50% among males and 37% among females. This result is in line with a previous large Italian cohort study, which showed higher rates of avoidable hospitalization among adults from countries with strong migratory pressure, especially among males (23), and with the results of a recent systematic review (21). These findings seem to confirm the lower recourse among immigrants to primary and specialist care (9). In fact, this indicator is based on the rationale that the lack of or delay in preventive, primary, and outpatient care for important health problems result in acute events that require hospitalization (21, 32).

The third health-care setting we evaluated was emergency care. In our study, the differences between Italians and immigrants in emergency department access were proportionally greater for codes indicating lesser severity (white and green) than for the most serious cases (yellow and red codes), confirming the greater inappropriateness of access among immigrants.

Immigrants, especially non-Europeans, access emergency departments to a greater extent than does the native population, especially in the initial period after arrival, probably due to their lack of familiarity with the health-care system of their host country (25). It should be pointed out that the hospital is the only or the main point of access to health-care services in many immigrants' country of origin (25, 33).

In Italy, all residents, including newly arrived immigrants, have the right to access primary care by registering with a general practitioner or a pediatrician. It can be hypothesized that many immigrants are unaware of this fundamental right, guaranteed by the universal health system, probably because navigating the complex bureaucracy, compounded by language barriers, makes the service inaccessible. Further confirmation is provided by the observation of higher access to emergency departments among immigrant females, very frequently of reproductive age, for obstetric and gynecological services (3, 25, 34).

In addition, access to the emergency department makes it possible to overcome some of the difficulties that immigrants encounter in primary care, such as doctors' limited office hours, which can represent an insurmountable barrier to accessing primary care for people who often work in extremely precarious conditions and without flexible work hours, as confirmed by the observation of emergency department access at off-hours and for less serious conditions. Experiences aimed at making primary care services more accessible could contribute to intercepting the health needs of immigrants while at the same time reducing the pressure on emergency departments (3, 10, 23, 33, 35).

A large body of literature has shown that immigrants risk unequal access to appropriate health care because they often face formal and informal barriers in accessing health services. Immigrants do not always have access to the services they need because of their legal status, but even in countries where access to health care is guaranteed to all immigrant populations, as it is in Italy, these individuals experience barriers raised by individual, sociocultural, economic, administrative, and political issues (17).

Structural barriers related to legal restrictions established according to the specific health-care model of each country may limit the entitlement to health care of the most vulnerable groups, including immigrants (36). Regarding informal barriers, health-care utilization may differ from that of the native population because of factors related to the migratory process. Newcomers in particular are less knowledgeable about how the health-care system is organized and how to navigate it (10).

Moreover, immigrants may generally have a more “utilitarian” concept of health, associated with the ability to work. Prevention activities, particularly those monitoring the evolution of chronic conditions, may not be considered a priority because they are not acknowledged as needs, at least in the early years in the host country (12).

The study findings show differences between Italians and immigrants in access to and outcomes of health care. From the moment from their arrival in the host country, immigrants tend to accumulate disadvantages in living and working conditions that reflect those typical of the lower social classes of the host population. The negative effects of these conditions on health are well-known and have been documented in all European countries (37). The social segregation mechanism, together with possible experiences of racial discrimination, lead to a phenomenon known as the “exhausted migrant effect,” which states that migrants eventually lose their initial health capital (38). Coherently with this theory, it has been shown that immigrants' health outcomes are mediated by social deprivation (39). Similarly, immigrants share with the lowest socioeconomic groups obstacles in accessing effective health care, even when no formal economic or legal barrier is in place.

Given the current situation in Italy, immigrants could become the least healthy segment of the population (22), as is the case in countries with a longer tradition of immigration (40). In particular, the socioeconomic shock induced by the COVID-19 pandemic in Italy as well as in other European countries may be a potential source of social exclusion and marginalization for the immigrant population, a condition that could affect their well-being or health status (41).

### Study Strengths and Limitations

The findings of our study are particularly meaningful because they are based on data from the MSHIP, which systematically collects and compares information on Italian and immigrant residents. These data are useful to developing healthcare intervention policies aimed at health equity.

However, the study has some limitations. First of all, the MSHIP currently has a coverage of less than half of the residents in Italy. More importantly, the macro areas of the country are not homogeneously represented, in particular due to the low coverage of Southern Italy. Secondly, the monitoring system targets only the resident population, without any possibility of evaluating undocumented immigrants.

## Conclusions

As immigrants cannot be considered a homogeneous group given their differing cultural backgrounds, historical roots, and ethnic features as well as differences in their health literacy, knowledge of how the health system works, financial aspects, and past experiences of care, public health policies should be based on culturally sensitive strategies to overcome barriers to health-care access (11, 21). One of the most potentially dangerous barriers is communication; when immigrants cannot adequately express themselves in or understand the language of the host country, they risk not knowing about available health and social support services, thereby increasing their risk of a delay in or lack of care (11, 21). Therefore, it is essential that professional interpreters be available in all settings and at all points of care (21).

An overall public health response based on the WHO's Health in All Policies is necessary; although not designed specifically for migrant health, it has an undeniable role in addressing social determinants of health (41). Policies aimed at improving socioeconomic conditions and promoting integration should include promotion of healthy lifestyles and appropriate access to health care, counteracting the emergence of health inequities in immigrant populations (42).

## Data Availability Statement

The data analyzed in this study is subject to the following licenses/restrictions: the data were collected using regional health information system databases. Each region has a policy about availability of any data. However, all relevant findings were produced using aggregated and not individual data. Requests to access these datasets should be directed to not applicable.

## Ethics Statement

Ethical review and approval was not required for the study on human participants in accordance with the local legislation and institutional requirements. Written informed consent for participation was not required for this study in accordance with the national legislation and the institutional requirements.

## MSHIP Group

*INMP:* Anteo Di Napoli, Alessandra Rossi, Martina Ventura, Raffaella Gaudio, Alessio Petrelli *Piedmont Region:* Teresa Spadea, Raffaella Rusciani, Luisa Mondo, Giuseppe Costa *Trento Province:* Laura Battisti, Pirous Fateh-Moghadam *Bolzano/Bozen Province:* Antonio Fanolla, Carla Melani *Emilia-Romagna Region:* Nicola Caranci, Letizia Bartolini, Laura Bonvicini, Serena Broccoli *Umbria Region:* Paola Casucci, Marcello De Giorgi, David Franchini *Tuscany Region:* Marco Lazzeretti, Caterina Silvestri, Fabio Voller, Simone Bartolacci, Mirko Monnini, Eleonora Fanti *Latium Region:* Laura Cacciani, Eleonora Trappolini, Alessandro Natali, Nera Agabiti, Marina Davoli *Basilicata Region*: Gabriella Cauzillo, Michele Recine, Anna Rita Lucia, Pierluigi Tramutoli, Enrico Barone, Pasquale Belviso, Rosalba Potenza, Mariangela Mininni *Sicily Region:* Achille Cernigliaro, Salvatore Scondotto, Elisa Eleonora Tavormina, Alessandra Vincenza Allotta.

## Author Contributions

ADN, MV, and AP took part in the conceptualization of the study, bibliographic research, development and implementation of methods, statistical analysis, and preparation of manuscript. TS, PGR, LBar, LBat, LC, NC, AC, MDG, AF, ML, MM, and CM took part in the conceptualization of the study, development and implementation of methods, and preparation of manuscript. All the authors could access and verify the data, approved the manuscript, and are responsible for the views expressed in it.

## Conflict of Interest

The authors declare that the research was conducted in the absence of any commercial or financial relationships that could be construed as a potential conflict of interest.

## Publisher's Note

All claims expressed in this article are solely those of the authors and do not necessarily represent those of their affiliated organizations, or those of the publisher, the editors and the reviewers. Any product that may be evaluated in this article, or claim that may be made by its manufacturer, is not guaranteed or endorsed by the publisher.
