# Supplementary material for: Barriers to Accessing Primary Care and Appropriateness of Healthcare Among Immigrants in Italy
Source: Front Public Health. 2022 Feb 9;10:817696. doi: 10.3389/fpubh.2022.817696 (PMC8864157; doi:10.3389/fpubh.2022.817696)
Supplement: Supplementary file 1 [file Table_1.docx]

**SUPPLEMENTARY MATERIAL**

**TABLE OF CONTENTS**

1. Table S1: indicators of maternal health care, by citizenship and region of residence. Italy, MSHIP, 2016-2017.
2. Table S2: avoidable hospitalizations, by gender, citizenship and region of residence. Italy, MSHIP, 2016-2017.
3. Table S3: access to the emergency care by triage code, citizenship and region of residence, men. Italy, MSHIP, 2016-2017.
4. Table S4: access to the emergency care by triage code, citizenship and region of residence, women. Italy, MSHIP, 2016-2017.

**Table S1: Indicators of maternal health care, by citizenship and region of residence. Italy, MSHIP, 2016-2017.**

| REGION OF RESIDENCE | Pregnancies | | During pregnancy | | | | | | | | | | | |
| --- | --- | --- | --- | --- | --- | --- | --- | --- | --- | --- | --- | --- | --- | --- |
|  |  |  | Less than 5 visits | | | | First visit at more than 12 weeks of gestational age | | | | Less than 2 ultrasounds | | | |
|  | Italians | Immigrants | Italians | | Immigrants | | Italians | | Immigrants | | Italians | | Immigrants | |
|  | N | | N | % | N | % | N | % | N | % | N | % | N | % |
| Piedmont | 43,391 | 16893 | 3,641 | 8.4 | 2,618 | 15.5 | 1,287 | 3.0 | 1,779 | 10.5 | 504 | 1.2 | 925 | 5.5 |
| Bolzano | 8,768 | 2,300 | 533 | 6.1 | 274 | 11.9 | 507 | 5.8 | 457 | 19.8 | 195 | 2.2 | 86 | 3.7 |
| Trento | 6,158 | 2,222 | 1,082 | 17.6 | 550 | 24.8 | 663 | 10.8 | 615 | 27.7 | 14 | 0.2 | 43 | 1.9 |
| Emilia-Romagna | 44,887 | 22,180 | 2,647 | 5.9 | 3,415 | 15.4 | 2,170 | 4.8 | 3,914 | 17.6 | 259 | 0.6 | 632 | 2.8 |
| Tuscany | 34,066 | 13,583 | 2,299 | 6.7 | 2,416 | 17.8 | 779 | 2.3 | 1,346 | 9.9 | 334 | 1.0 | 423 | 3.1 |
| Umbria | 9,951 | 2,767 | 193 | 1.9 | 168 | 6.1 | 171 | 1.7 | 167 | 6.0 | 55 | 0.6 | 113 | 4.1 |
| Latium | 70,379 | 19,257 | - | - | - | - | 3,579 | 5.1 | 1,654 | 8.6 | - | - | - | - |
| Basilicata | 4,122 | 11 | 516 | 12.5 | 4 | 36.4 | 121 | 2.9 | 3 | 27.3 | 54 | 1.3 | - | - |
| Sicily | 37,289 | 2,929 | 5,120 | 13.7 | 814 | 27.8 | 639 | 1.7 | 295 | 10.1 | 514 | 1.4 | 193 | 6.6 |
| TOTAL | 259,011 | 82,142 | 16,031 | 8.5 | 10,260 | 16.3 | 9,916 | 3.8 | 10,229 | 12.5 | 1,929 | 1.0 | 2,415 | 3.8 |

**Table S2: Avoidable hospitalizations, by gender, citizenship and region of residence. Italy, MSHIP, 2016-2017.**

| AVOIDABLE HOSPITALIZATION  (20-64 years) | Men | | | | | | Women | | | | | |
| --- | --- | --- | --- | --- | --- | --- | --- | --- | --- | --- | --- | --- |
|  | Italians | | | Immigrants | | | Italians | | | Immigrants | | |
|  | N | Age standardized Rate *1000 | 95%CI | N | Age standardized Rate *1000 | 95%CI | N | Age standardized Rate *1000 | 95%CI | N | Age standardized Rate *1000 | 95%CI |
| Piedmont | 2,214 | 0.90 | (0.86-0.94) | 217 | 1.28 | (1.10-1.47) | 1,165 | 0.49 | (0.46-0.52) | 143 | 0.57 | (0.47-0.66) |
| Bolzano | 396 | 1.38 | (1.24-1.51) | 56 | 2.56 | (1.84-3.27) | 126 | 0.47 | (0.39-0.55) | 26 | 0.89 | (0.54-1.25) |
| Trento | 358 | 1.18 | (1.05-1.30) | 34 | 1.88 | (1.21-2.55) | 210 | 0.73 | (0.63-0.83) | 31 | 1.09 | (0.70-1.49) |
| Emilia-Romagna | 5,502 | 2.27 | (2.21-2.33) | 766 | 3.34 | (3.08-3.59) | 2,562 | 1.11 | (1.07-1.15) | 492 | 1.84 | (1.66-2.01) |
| Tuscany | 3,069 | 1.50 | (1.45-1.55) | 293 | 1.72 | (1.51-1.93) | 1,244 | 0.63 | (0.59-0.66) | 174 | 0.65 | (0.55-0.75) |
| Umbria | 508 | 1.03 | (0.94-1-12) | 51 | 1.21 | (0.85-1.57) | 285 | 0.58 | (0.51-0.64) | 44 | 0.66 | (0.46-0.86) |
| Latium | 4,580 | 1.45 | (1.41-1.51) | 738 | 2.39 | (2.21-2.58) | 2,083 | 0.66 | (0.63-0.68) | 359 | 0.83 | (0.74-0.91) |
| Basilicata | 600 | 3.55 | (3.26-3.83) | 4 | 0.69 | (0.10-1.28) | 242 | 1.45 | (1.27-1.63) | 16 | 2.18 | (1.08-3.27) |
| Sicily | 986 | 0.72 | (0.67-0.76) | 17 | 0.38 | (0.19-0.56) | 510 | 0.36 | (0.33-0.39) | 17 | 0.33 | (0.17-0.48) |
| TOTAL | 18,213 | 1.43 | (1.41-1.45) | 2,176 | 2.14 | (2.04-2.23) | 8,427 | 0.67 | (0.66-0.69) | 1,302 | 0.92 | (0.87-0.97) |

**Table S3: access to the emergency care by triage code, citizenship and region of residence, men. Italy, MSHIP, 2016-2017.**

| EMERGENCY CARE - Men | TOTAL | | | WHITE | | | GREEN | | | YELLOW | | | RED | | |
| --- | --- | --- | --- | --- | --- | --- | --- | --- | --- | --- | --- | --- | --- | --- | --- |
| REGION | N | Age standardized Rate *1000 | 95%CI | N | Age standardized Rate *1000 | 95%CI | N | Age standardized Rate *1000 | 95%CI | N | Age standardized Rate *1000 | 95%CI | N | Age standardized Rate *1000 | 95%CI |
| PIEDMONT |  |  |  |  |  |  |  |  |  |  |  |  |  |  |  |
| Italians | 1,505,896 | 341.22 | (340.54-341.89) | 216,340 | 55.21 | (54.94-55.49) | 1,034,292 | 246.08 | (245.51-246.66) | 237,309 | 37.94 | (37.72-38.17) | 17,955 | 1.98 | (1.93-2.03) |
| Immigrants | 203,637 | 465.00 | (462.60-467.40) | 50,709 | 117.18 | (115.97-118.39) | 135,001 | 305.3 | (303.38-307.22) | 16,942 | 40.25 | (39.49-41.00) | 985 | 2.27 | (2.08-2.46) |
| BOLZANO |  |  |  |  |  |  |  |  |  |  |  |  |  |  |  |
| Italians | 214,267 | 359.62 | (357.73-361.51) | 10,136 | 17.52 | (17.10-17.94) | 129,486 | 234.61 | (233.09-236.13) | 72,602 | 105.35 | (104.32-106.37) | 2,043 | 2.15 | (2.00-2.29) |
| Immigrants | 49,665 | 536.31 | (528.90-543.73) | 3,028 | 48.79 | (46.56-51.03) | 30,039 | 356.59 | (350.59-362.52) | 16,080 | 128.60 | (124.90-132.31) | 518 | 2.33 | (1.81-2.84) |
| TRENTO |  |  |  |  |  |  |  |  |  |  |  |  |  |  |  |
| Italians | 195,165 | 298.14 | (296.41-299.86) | 40,036 | 69.23 | (68.40-70.06) | 121,137 | 192.15 | (190.76-193.53) | 31,818 | 35.27 | (34.67-35.86) | 2,174 | 1.50 | (1.38-1.62) |
| Immigrants | 24,167 | 409.51 | (402.64-416.37) | 6,171 | 109.09 | (105.65-112.53) | 15,008 | 256.14 | (250.73-261.55) | 2,847 | 42.29 | (39.90-44.67) | 141 | 1.99 | (1.44-2.54) |
| EMILIA-ROMAGNA |  |  |  |  |  |  |  |  |  |  |  |  |  |  |  |
| Italians | 1,602,806 | 354.97 | (354.28-355.66) | 203,453 | 50.51 | (50.25-50.77) | 1,045,937 | 251.45 | (250.87-252.04) | 317,124 | 49.20 | (48.94-49.45) | 36,292 | 3.81 | (3.74-3.88) |
| Immigrants | 262,573 | 464.34 | (462.21-466.47) | 52,000 | 91.19 | (90.26-92.13) | 178,427 | 314.38 | (312.65-316.11) | 29,583 | 54.12 | (53.34-54.90) | 2,563 | 4.65 | (4.41-4.89) |
| TUSCANY |  |  |  |  |  |  |  |  |  |  |  |  |  |  |  |
| Italians | 978,436 | 249.62 | (248.99-250.26) | 67,735 | 22.39 | (22.20-22.58) | 613,951 | 177.23 | (176.69-177.76) | 269,126 | 46.50 | (46.23-46.77) | 27,624 | 3.50 | (3.43-3.58) |
| Immigrants | 101,676 | 272.22 | (270.33-274.10) | 14,052 | 37.00 | (36.33-37.67) | 69,142 | 183.8 | (182.27-185.32) | 17,085 | 47.60 | (46.77-48.43) | 1,397 | 3.82 | (3.58-4.06) |
| UMBRIA |  |  |  |  |  |  |  |  |  |  |  |  |  |  |  |
| Italians | 298,530 | 317.13 | (315.65-318.59) | 34,307 | 41.40 | (40.87-41.92) | 204,458 | 233.31 | (232.05-234.56) | 55,868 | 40.45 | (39.94-40.97) | 3,897 | 1.98 | (1.86-2.09) |
| Immigrants | 29,776 | 324.12 | (319.70-328.54) | 4,600 | 50.42 | (48.68-52.16) | 21,294 | 231.21 | (227.52-234.91) | 3,677 | 40.31 | (38.67-41.95) | 205 | 2.17 | (1.79-2.56) |
| LATIUM |  |  |  |  |  |  |  |  |  |  |  |  |  |  |  |
| Italians | 1,648,334 | 283.52 | (282.99-284.04) | 62,787 | 11.89 | (11.78-11.99) | 1,071,775 | 206.21 | (205.76-206.65) | 457,976 | 59.78 | (59.54-60.02) | 55,796 | 5.65 | (5.57-5.72) |
| Immigrants | 237,669 | 314.37 | (312.79-315.95) | 19,052 | 24.91 | (24.48-25.35) | 167,095 | 221.63 | (220.32-222.93) | 45,997 | 61.01 | (60.28-61.74) | 5,525 | 6.82 | (6.57-7.07) |
| BASILICATA |  |  |  |  |  |  |  |  |  |  |  |  |  |  |  |
| Italians | 78,965 | 254.02 | (251.88-256.16) | 7,708 | 28.24 | (27.53-28.95) | 53,107 | 185.97 | (184.13-173.60) | 17,317 | 38.62 | (37.79-39.45) | 833 | 1.19 | (1.05-1.34) |
| Immigrants | 3,038 | 317.04 | (303.19-330.88) | 388 | 36.10 | (31.71-40.50) | 2,270 | 237.22 | (225.27-249.16) | 364 | 41.70 | (36.42-46.99) | 16 | 2.01 | (0.74-3.28) |
| SICILY |  |  |  |  |  |  |  |  |  |  |  |  |  |  |  |
| Italians | 800,723 | 336.68 | (335.94-337.43) | 50,417 | 21.38 | (21.19-21.57) | 534,316 | 222.48 | (221.88-223.09) | 201,675 | 86.56 | (86.18-86.95) | 14,315 | 6.26 | (6.15-6.36) |
| Immigrants | 21,413 | 249.20 | (202.64-295.76) | 2,510 | 25.09 | (13.33-36.85) | 14,779 | 148.48 | (88.65-208.31) | 3,923 | 71.45 | (65.57-77.33) | 201 | 4.18 | (3.20-5.16) |
| TOTAL |  |  |  |  |  |  |  |  |  |  |  |  |  |  |  |
| Italians | 7,323,122 | 309.21 | (308.93-309.48) | 692,919 | 32.70 | (32.61-32.79) | 4,808,459 | 221.32 | (221.08-221.55) | 1,660,815 | 51.51 | (51.40-51.62) | 160,929 | 3.68 | (3.65-3.71) |
| Immigrants | 933,614 | 371.82 | (370.91-372.73) | 152,510 | 61.98 | (61.62-62.35) | 633,055 | 252.74 | (251.99-253.48) | 136,498 | 52.69 | (52.33-53.06) | 11,551 | 4.41 | (4.30-4.51) |

**Table S4: access to the emergency care by triage code, citizenship and region of residence, women. Italy, MSHIP, 2016-2017.**

| EMERGENCY CARE - Women | TOTAL | | | WHITE | | | GREEN | | | YELLOW | | | RED | | |
| --- | --- | --- | --- | --- | --- | --- | --- | --- | --- | --- | --- | --- | --- | --- | --- |
| REGION | N | Age standardized Rate *1000 | 95%CI | N | Age standardized Rate *1000 | 95%CI | N | Age standardized Rate *1000 | 95%CI | N | Age standardized Rate *1000 | 95%CI | N | Age standardized Rate *1000 | 95%CI |
| PIEDMONT |  |  |  |  |  |  |  |  |  |  |  |  |  |  |  |
| Italians | 1,538,749 | 344.61 | (343.92-345.30) | 219,036 | 54.75 | (54.47-55.02) | 1,085,322 | 257.17 | (256.57-257.77) | 219,933 | 31.59 | (31.39-31.80) | 14,458 | 1.10 | (1.06-1.14) |
| Immigrants | 229,273 | 456.00 | (453.91-458.09) | 49,168 | 101.01 | (100.01-102.00) | 160,048 | 315.87 | (314.14-317.59) | 19,365 | 37.96 | (37.34-38.58) | 692 | 1.17 | (1.06-1.28) |
| BOLZANO |  |  |  |  |  |  |  |  |  |  |  |  |  |  |  |
| Italians | 196,463 | 315.57 | (313.76-317.39) | 8,721 | 14.87 | (14.48-15.27) | 115,116 | 199.05 | (197.61-200.49) | 71,306 | 100.60 | (99.57-101.63) | 1,320 | 1.05 | (0.94-1.15) |
| Immigrants | 46,834 | 475.62 | (469.16-482.08) | 1,907 | 29.15 | (27.54-30.76) | 28,144 | 299.89 | (294.75-305.03) | 16,505 | 145.21 | (141.67-148.75) | 278 | 1.37 | (1.01-1.73) |
| TRENTO |  |  |  |  |  |  |  |  |  |  |  |  |  |  |  |
| Italians | 191,703 | 288.19 | (286.45-289.93) | 34,460 | 59.74 | (58.95-60.52) | 125,187 | 198.86 | (197.41-200.31) | 30,225 | 28.81 | (28.26-29.36) | 1,831 | 0.78 | (0.69-0.87) |
| Immigrants | 26,837 | 403.30 | (397.50-409.10) | 5,553 | 90.57 | (87.81-93.33) | 18,071 | 269.63 | (264.92-274.34) | 3,120 | 42.20 | (40.26-44.13) | 93 | 0.90 | (0.61-1.18) |
| EMILIA-ROMAGNA |  |  |  |  |  |  |  |  |  |  |  |  |  |  |  |
| Italians | 1,607,358 | 341.15 | (340.46-341.83) | 206,071 | 51.72 | (51.45-51.99) | 1,061,484 | 247.23 | (246.64-247.81) | 306,277 | 39.92 | (39.69-40.16) | 33,526 | 2.28 | (2.23-2.34) |
| Immigrants | 275,597 | 457.01 | (455.03-458.99) | 49,927 | 83.19 | (82.35-84.03) | 194,123 | 320.03 | (318.39.321.67) | 29,750 | 51.05 | (50.35-51.75) | 1,797 | 2.74 | (2.57-2.91) |
| TUSCANY |  |  |  |  |  |  |  |  |  |  |  |  |  |  |  |
| Italians | 1,007,045 | 237.95 | (237.32-238.57) | 59,205 | 19.34 | (19.16-19.52) | 646,017 | 176.12 | (175.58-176.66) | 277,623 | 40.63 | (40.37-40.88) | 24,200 | 1.86 | (1.80-1.91) |
| Immigrants | 111,762 | 250.77 | (249.20-252.35) | 10,991 | 25.89 | (25.39-26.40) | 79,766 | 179.98 | (178.66-181.31) | 20,035 | 42.94 | (42.28-43.60) | 970 | 1.95 | (1.81-2.10) |
| UMBRIA |  |  |  |  |  |  |  |  |  |  |  |  |  |  |  |
| Italians | 277,992 | 277.84 | (276.46-279.21) | 30,855 | 37.40 | (36.90-37.91) | 191,624 | 205.75 | (204.57-206.94) | 52,069 | 33.61 | (33.13-34.09) | 3,444 | 1.07 | (0.99-1.16) |
| Immigrants | 34,238 | 275.85 | (271.56-279.15) | 4,652 | 38.10 | (36.87-39.33) | 24,807 | 201.10 | (198.29-203.91) | 4,623 | 35.55 | (34.36-36.74) | 156 | 1.11 | (0.89-1.32) |
| LATIUM |  |  |  |  |  |  |  |  |  |  |  |  |  |  |  |
| Italians | 1,660,736 | 276.32 | (275.81-276.84) | 60,942 | 11.52 | (11.41-11.62) | 1,087,758 | 204.05 | (203.60-204.49) | 467,296 | 57.61 | (57.37-57.85) | 44,740 | 3.15 | (3.10-3.21) |
| Immigrants | 271,157 | 323.91 | (322.49-325.34) | 16,379 | 20.20 | (19.83-20.56) | 192,438 | 233.69 | (232.48-234.90) | 58,275 | 66.00 | (65.36-66.65) | 4,065 | 4.02 | (3.86-4.19) |
| BASILICATA |  |  |  |  |  |  |  |  |  |  |  |  |  |  |  |
| Italians | 74,812 | 234.40 | (232.32-236.49) | 6,098 | 21.53 | (20.90-22.16) | 50,642 | 175.40 | (173.60-177.20) | 17,365 | 36.79 | (35.97-37.61) | 707 | 0.68 | (0.57-0.79) |
| Immigrants | 3,817 | 335.08 | (323.62-346.54) | 340 | 32.21 | (28.52-35.89) | 2,755 | 242.56 | (232.83-252.28) | 707 | 59.25 | (54.49-64.01) | 15 | 1.06 | (0.38-1.75) |
| SICILY |  |  |  |  |  |  |  |  |  |  |  |  |  |  |  |
| Italians | 795,579 | 313.44 | (312.74-314.13) | 48,044 | 18.98 | (18.81-19.15) | 535,149 | 208.62 | (208.06-209.19) | 200,898 | 81.04 | (80.69-81.40) | 11,488 | 4.79 | (4.70-4.87) |
| Immigrants | 25,147 | 298.74 | (250.82-346.67) | 2,116 | 22.52 | (10.76-34.28) | 17,524 | 179.25 | (116.96-241.55) | 5,306 | 91.40 | (85.52-97.28) | 201 | 5.57 | (4.59-6.55) |
| TOTAL |  |  |  |  |  |  |  |  |  |  |  |  |  |  |  |
| Italians | 7,350,437 | 299.44 | (299.17-299.72) | 673,432 | 31.37 | (31.28-31.46) | 4,898,299 | 219.38 | (219.14-219.61) | 1,642,992 | 46.62 | (46.51-46.72) | 135,714 | 2.08 | (2.06-2.10) |
| Immigrants | 1,024,662 | 365.30 | (364.49-366.11) | 141,033 | 52.91 | (52.61-53.22) | 717,676 | 257.63 | (256.96-258.31) | 157,686 | 52.27 | (51.96-52.58) | 8,267 | 2.49 | (2.42-2.56) |
